# Microbiota–host crosstalk in ulcerative colitis: mechanistic evidence and translational prospects for traditional Chinese medicine

**DOI:** 10.3389/fphar.2026.1956353

**Published:** 2026-10-09

**Authors:** Jing Chen, Jing Du, Baoxi Mu, Jie Zhang, Guangbin Shang, Xiaojun Yan

**Affiliations:** 1 Jiangxi University of Chinese Medicine, Nanchang, China; 2 The Affiliated Hospital of Jiangxi University of Chinese Medicine, Nanchang, China

**Keywords:** gut microbiota, microbial metabolites, traditional Chinese medicine, translational pharmacology, ulcerative colitis

## Abstract

Ulcerative colitis (UC) is sustained by reciprocal interactions among intestinal dysbiosis, altered microbial metabolism, epithelial barrier dysfunction, and mucosal immune activation. This narrative review examines that microbiota–metabolism–barrier–immune network and evaluates how traditional Chinese medicine (TCM) may influence it. Human multi-omics studies indicate that UC-associated dysbiosis is more reproducible at the level of microbial function and metabolite output than at the level of individual taxa. Experimental studies further implicate short-chain fatty acids, bile acids, tryptophan-derived indoles, arginine, and choline-related metabolites in epithelial and immune regulation. Most evidence for TCM-mediated microbiota modulation, however, comes from chemically induced experimental colitis, organoid, or cell systems; human interventional evidence remains limited. We therefore grade mechanistic evidence from association through microbiota dependence, candidate strain/metabolite sufficiency, host-target validation, and integrated causal-chain validation. This distinction avoids treating genus-level relative-abundance changes as proof of microbial benefit or mediation. Emerging clinical trials of indigo naturalis, Curcumin–QingDai, *Andrographis paniculata* extract, curcumin, and selected multicomponent Chinese herbal therapies provide short-term efficacy signals, but results are preparation- and dose-dependent and do not yet establish microbiota-mediated clinical efficacy. Translation will require chemically standardized products, rigorous safety surveillance, strain- and function-resolved microbiome measurements, explicit experiments separating direct herbal actions from microbiota-dependent effects, and clinical trials incorporating endoscopic, histological, pharmacokinetic, microbial, metabolic, and long-term safety outcomes.

## 1 Introduction

Ulcerative colitis (UC) is a chronic inflammatory bowel disease of the colorectal mucosa with a relapsing–remitting course (Le Berre et al., 2023). The disease can cause persistent bleeding and diarrhea, hospitalization, colectomy, and inflammation-associated colorectal cancer (Sebastian et al., 2014). Current treatment includes aminosalicylates, corticosteroids, immunomodulators, small molecules, and biologics (Inflammatory Bowel Disease Group of Chinese Society of Gastroenterology of Chinese Medical Association and Inflammatory Bowel Disease Quality Control Center of China, 2024; Gisbert et al., 2026), yet primary non-response, secondary loss of response, treatment toxicity, and unequal access remain clinically relevant.

UC arises from interactions among genetic susceptibility, environmental exposure, epithelial barrier defects, immune dysregulation, and disruption of the intestinal microecosystem (Le Berre et al., 2023; Ramos and Papadakis, 2019). The gut microbiota contributes to nutrient processing, metabolite production, colonization resistance, barrier maintenance, and immune calibration. In UC, dysbiosis is both a potential driver and a consequence of inflammation: community structure changes, microbial functions are reprogrammed, and host responses further reshape the ecological niche (Franzosa et al., 2019). Microbial abnormalities are detectable in newly diagnosed, treatment-naive disease, and selected microbial or metabolic features have been associated with activity, therapeutic response, and subsequent relapse (Lloyd-Price et al., 2019; Halfvarson et al., 2017; Schirmer et al., 2018; Schirmer et al., 2024). These observations justify a mechanistic focus on the microbiota–metabolism–barrier–immune axis, but they do not by themselves establish that a specific taxon causes disease or mediates treatment efficacy.

TCM has a long history of use in UC and commonly describes patterns such as spleen deficiency, damp-heat, toxin accumulation, and blood stasis (Li JX. et al., 2024; Spleen and Stomach Diseases Branch and China Association of Chinese Medicine, 2024). Here, “spleen deficiency” denotes a TCM functional construct used to describe a cluster of digestive and constitutional features; it does not imply disease or failure of the anatomical spleen. TCM syndrome constructs and biomedical mechanisms are not interchangeable. Their pharmacological value is therefore best tested as prespecified stratification hypotheses rather than assumed to correspond one-to-one with microbial or immune pathways. This review asks which components of the UC microecological network are pharmacologically modifiable and how convincingly the microbiota has been shown to mediate reported herbal effects.

### 1.1 Scope, literature identification, and analytical framework

This is a narrative, mechanism-focused review rather than a systematic review. To improve transparency, the literature was identified by targeted searches centered on PubMed/MEDLINE and cross-reference checking, supplemented by Chinese-language literature already represented in the evidence base. Searches for this revision were updated through August 2026, with no lower date limit; English- and Chinese-language peer-reviewed reports were considered. Search concepts combined ulcerative colitis or experimental colitis with gut microbiota/microbiome, metagenomics, metabolomics, short-chain fatty acids, bile acids, tryptophan/indoles, arginine, choline/TMAO, and traditional Chinese medicine/herbal formula/decoction terms. Peer-reviewed human UC studies and mechanistically informative experimental-colitis studies were prioritized; conference abstracts, unpublished material, and studies unrelated to intestinal inflammation were excluded. “Representative” studies in the tables were selected to capture different formulation classes and levels of causal validation rather than to provide exhaustive coverage. Because no registered protocol, duplicate screening process, or formal systematic risk-of-bias synthesis was prespecified, this narrative approach is vulnerable to selection and publication bias and should not be interpreted as a systematic estimate of effect size.

Mechanistic evidence is graded at five levels: G1, association, when microbial and host changes co-occur; G2, microbiota dependence/transferability, when antibiotic depletion, germ-reduced conditions, or microbiota transfer alters efficacy; G3, candidate sufficiency, when a defined strain or metabolite reproduces or rescues part of an effect; G4, host-target validation, when receptor antagonism, genetic perturbation, or direct target-engagement verifies the receiving host pathway; and G5, an integrated causal chain that combines microbiota dependence, candidate manipulation, and host-target perturbation. Organoid or cell experiments without target perturbation are treated as supportive host-functional assays rather than G4 validation. Likewise, strain or metabolite supplementation demonstrates partial sufficiency, not mediation or necessity by itself. This hierarchy is applied throughout the review.

## 2 Gut microbiota dysbiosis in ulcerative colitis

### 2.1 Multi-omics features of dysbiosis

UC-associated dysbiosis cannot be reduced to the expansion of a few “harmful” organisms and the loss of a few “beneficial” ones. Multi-omics studies identify coordinated changes in bacterial communities, the mycobiome and virome, microbial functional genes, metabolite outputs, and host molecular responses (Lloyd-Price et al., 2019; Mills et al., 2022; Scanu et al., 2024; Akiyama et al., 2024). Integrated analyses have reported bacterial–fungal–metabolic signatures that distinguish UC from health (Scanu et al., 2024), yet cross-cohort comparisons show substantial heterogeneity in the direction and magnitude of individual taxonomic changes. Community-level configuration, functional pathways, and metabolite profiles are generally more reproducible than single taxa (Ning et al., 2023; Abdel-Rahman and Morgan, 2023). The more informative question is therefore how an ecological network that supports mucosal homeostasis is reorganized into one that tolerates and reinforces inflammation.

Reduced α diversity is frequently reported in UC, and β-diversity profiles often differ from those of healthy controls (Akiyama et al., 2024; Abdel-Rahman and Morgan, 2023). These metrics are descriptive rather than inherently mechanistic: their magnitude depends on sample type, disease activity, medication exposure, diet, sequencing depth, and analytical pipeline. Akiyama et al. found that UC involved not only reduced bacterial diversity and altered bacterial composition but also disrupted interactions among bacteriophages, fungi, and other microbial components (Akiyama et al., 2024). Jangi et al. (2024a) observed greater fecal *Candida* abundance during active than quiescent UC, with partial reversal as inflammation resolved. Such findings support disease-state associations, but longitudinal and functional experiments are needed before diversity or fungal abundance can be treated as a causal or predictive biomarker.

Several UC and inflammatory bowel disease cohorts have reported enrichment of *Ruminococcus gnavus*, Enterobacteriaceae, and *Escherichia coli*, together with depletion of butyrate-producing commensals such as *Faecalibacterium prausnitzii* and *Roseburia intestinalis* (Lloyd-Price et al., 2019; Mills et al., 2022; Machiels et al., 2014). The direction of change for individual genera or species is not consistent across populations (Abdel-Rahman and Morgan, 2023), and taxonomic labels may conceal strain-level functional differences. A more defensible interpretation is that homeostasis-supporting microbial networks are weakened while organisms adapted to oxidative stress, altered nutrient availability, and inflammatory metabolites gain an ecological advantage. This shift is likely context dependent rather than a universal taxonomic signature of UC.

Functional analyses provide a stronger bridge to pathogenesis than taxonomy alone. Cross-cohort metagenomic studies have identified enrichment of lipopolysaccharide biosynthesis, two-component systems, and other inflammation-associated pathways, together with reduced propionate metabolism, glycolysis, and selected amino acid biosynthetic functions (Ning et al., 2023). UC-focused studies also report depletion of mucosal butyrate producers and lower predicted butyrate-synthesis capacity during active disease (Jangi et al., 2024b). At the KEGG ortholog level, Akiyama et al. (2024) observed reduced N-glycan biosynthesis and glycosaminoglycan-degradation genes and increased peptidoglycan-biosynthesis genes in inflammatory bowel disease. These findings indicate a loss of homeostasis-supporting metabolic capacity, although pathway prediction from sequencing data should be distinguished from direct metabolite measurement and flux analysis.

Functional validation has begun to clarify why abundance alone can be misleading. Mills et al. (2022) identified excessive *Bacteroides vulgatus*-derived protease activity in a subset of patients with active UC. Broad-spectrum protease inhibition reduced *B. vulgatus*-induced epithelial dysfunction *in vitro* and attenuated colitis in IL-10-deficient mice mono-colonized with *B. vulgatus* or colonized with fecal microbiota from patients with high-protease UC. The study linked a microbial product to a host phenotype and an intervention response, illustrating that strain function and secreted effectors may be more informative than relative abundance.

Dysbiosis also reflects altered host selection pressures. In treatment-naive children with UC, microbial, epigenetic, and transcriptomic features were associated with subsequent relapse, and a combined microbiome–epigenome model outperformed either data layer alone (Kulecka et al., 2025). Relapse-associated organisms were linked to interferon signaling, innate immune activation, and epithelial barrier programs. UC mucosa also shows reduced SLC16A1 expression, impaired MCT1-mediated butyrate transport, and diminished butyrate oxidation (De Preter et al., 2012). Thus, reduced microbial production and defective host utilization can converge on the same metabolic deficit. UC dysbiosis is best understood as coupled ecological and host reorganization rather than as an isolated microbial lesion.

### 2.2 Microbiota-driven pathological mechanisms in ulcerative colitis

#### 2.2.1 Metabolic dysregulation

Five metabolic axes illustrate how microbial dysfunction can be translated into epithelial and immune injury: short-chain fatty acids (SCFAs), bile acids, tryptophan-derived indoles, arginine, and choline-related metabolites. These pathways are interconnected, vary by intestinal compartment and disease state, and can be influenced by diet, host enzymes, and medication. They should therefore be interpreted as dynamic networks rather than independent linear pathways.

##### 2.2.1.1 Short-chain fatty acid axis

Acetate, propionate, and butyrate are produced largely through microbial fermentation of dietary substrates and support colonocyte metabolism, barrier integrity, and immune regulation (Parada Venegas et al., 2019). UC is associated with depletion of several SCFA-producing taxa and reduced biosynthetic capacity (Machiels et al., 2014). Restoration of SCFA-associated communities increased fecal SCFAs and alleviated experimental colitis (Shen et al., 2026). In Caco-2 cells, butyrate-dependent vitamin C accumulation increased antioxidant capacity and reduced lipopolysaccharide-induced barrier injury. A randomized, double-blind trial reported that sodium butyrate (600 mg/day for 12 weeks) added to standard therapy reduced fecal calprotectin and high-sensitivity C-reactive protein and improved quality of life in mild-to-moderately active UC (Firoozi et al., 2024). A seven-strain SCFA-producing consortium and its culture supernatant also improved barrier function and shifted macrophages away from a CD86-positive inflammatory phenotype in DSS colitis (Zhao H. et al., 2024). Together, these data support SCFA insufficiency as a mechanistic link, while also showing that metabolite supplementation, microbial restoration, and host receptor responsiveness are not equivalent interventions.

##### 2.2.1.2 Bile acid axis

Gut microorganisms deconjugate and transform primary bile acids into secondary bile acids, including deoxycholic acid, lithocholic acid, and ursodeoxycholic acid. These metabolites signal through FXR, TGR5, PXR, and related pathways to influence epithelial integrity and mucosal immunity. UC cohorts show lower concentrations of several fecal secondary bile acids and relative accumulation of primary bile acids (Yang Z. H. et al., 2021), consistent with depletion of taxa involved in bile acid transformation. The non-tumorigenic FGF19 analog M52 altered the bile acid pool, enriched selected commensals, and reduced DSS colitis; attenuation of these effects in FXR-deficient mice supported an FXR-dependent mechanism (Gadaleta et al., 2020). Exogenous lithocholic acid also improved epithelial barrier function through PXR activation and suppression of TLR4/NF-κB/NLRP3 signaling (Liu et al., 2025a). These studies implicate receptor-active bile acid pools rather than a single bile acid concentration as the relevant pharmacological endpoint.

##### 2.2.1.3 Tryptophan–indole–AhR axis

Microbial metabolism converts tryptophan into indole derivatives that can activate the aryl hydrocarbon receptor (AhR), a regulator of epithelial repair and mucosal immunity. A tryptophan-rich diet reduced DSS colitis in mice (Scott et al., 2020). In epithelial-cell screening, indole-3-ethanol, indole-3-pyruvic acid, and indole-3-carboxaldehyde reduced TNF-α-induced hyperpermeability, preserved apical junctional complexes, suppressed TNFR1/NF-κB signaling, and increased IL-10 receptor expression. Pharmacological or genetic disruption of AhR attenuated these effects. Indole-3-carboxaldehyde also increased ZO-1 and occludin and reduced inflammatory signaling through TLR/NF-κB/p38 (Liu et al., 2023). The pathway is therefore supported by metabolite supplementation and receptor-level validation, although human dose–exposure relationships remain poorly defined.

##### 2.2.1.4 Arginine axis

L-Arginine participates in nitric oxide synthesis, polyamine metabolism, epithelial repair, and immune regulation. In active UC, colonic tissue can show low L-arginine, high L-citrulline, reduced SLC7A2 and ARG1, and increased NOS2 and ARG2, consistent with impaired uptake and accelerated local consumption (Brosnan and Brosnan, 2004; Coburn et al., 2016). In contrast, a multicenter multi-omics study reported higher serum and fecal L-arginine, a positive association with Mayo scores, and increased colonic ASS1; L-arginine supplementation or ASS1 overexpression worsened experimental colitis, whereas ASS1 inhibition was protective (Liu et al., 2025b). ARG1 deletion altered luminal polyamines and microbial ecology and accelerated resolution, and microbiota transfer reproduced part of that phenotype (Baier et al., 2020). These findings are not necessarily mutually exclusive: mucosal tissue, luminal contents, and circulating blood represent different metabolic compartments, and the direction of change may vary with inflammatory activity, substrate intake, host transport, microbial consumption, and treatment exposure. A patient could therefore have local mucosal depletion despite luminal or systemic accumulation. The current evidence does not support phase-independent arginine supplementation or depletion; longitudinal, compartment-resolved flux studies are needed before arginine can be treated as a stable UC biomarker or therapeutic target.

##### 2.2.1.5 Choline–TMAO axis

Microbial choline trimethylamine lyases convert choline to trimethylamine (TMA), which hepatic flavin-containing monooxygenase three oxidizes to trimethylamine N-oxide (TMAO) (Romano et al., 2015). Only a minority of intestinal microorganisms carry TMA-producing genes, but community composition and diet can markedly influence pathway activity (Rath et al., 2017; Arias et al., 2020; Mitchell et al., 2019; Haas et al., 2025). UC mucosa contains less phosphatidylcholine, a key component of the mucus barrier, and microbial phosphatidylcholine degradation may further reduce host choline availability (Ehehalt et al., 2004; Braun et al., 2009). Experimental TMAO exposure has been associated with impaired autophagy-related proteins, NLRP3 activation, oxidative stress, tight-junction disruption, and PERK–eIF2α–ATF4–CHOP signaling (Yue et al., 2017; Chen et al., 2019). Conversely, a methionine- and choline-deficient diet worsened DSS colitis in a microbiota-dependent manner and reduced several SCFAs (Liu M. T. et al., 2024). The available evidence therefore does not support a simple model in which higher TMAO alone drives UC. A more plausible interpretation is that dysbiosis perturbs choline allocation, phosphatidylcholine availability, TMA production, and downstream stress responses in a context-dependent manner.

#### 2.2.2 Intestinal barrier disruption

The intestinal barrier is not a single structure but a coordinated system comprising the mucus layer, epithelial junctions, antimicrobial programs, and resident immune cells. Dysbiosis can damage each component, while barrier failure exposes the mucosa to microbial products and changes the habitat in ways that further select for inflammation-adapted organisms.

The mucus layer is organized around MUC2 and contains a dense inner compartment that normally excludes bacteria and a looser outer compartment colonized by commensals (Johansson et al., 2008). Excessive microbial use of MUC2 glycans can thin and increase the penetrability of the inner layer (Desai et al., 2016). Persistent stimulation may also drive compensatory mucus secretion followed by goblet-cell depletion (Birchenough et al., 2016; Cornick et al., 2019; Yao et al., 2021). Human UC tissue shows reduced MUC2 synthesis, altered mucus architecture, increased penetrability, and bacterial encroachment (Van Klinken et al., 1999; van der Post et al., 2019; Johansson et al., 2014). These observations support mucus failure as an early interface between ecological disturbance and epithelial inflammation, but the relative contributions of reduced synthesis, altered glycosylation, proteolysis, and accelerated turnover remain unresolved.

Tight-junction disruption provides a second route to increased permeability. *B. vulgatus*-derived proteases reduce transepithelial electrical resistance, and protease inhibition limits epithelial damage *in vitro* and in mono-colonized IL-10-deficient mice (Mills et al., 2022). Adherent-invasive *E. coli* LF82 also reduces ZO-1 and claudin-1 expression, alters junctional localization, and increases TNF-α and IL-17 (Sha et al., 2024). These examples show that barrier injury may arise from strain-specific effectors rather than from broad taxonomic imbalance alone.

Subepithelial resident macrophages contribute to local containment and tissue repair. Jiang et al. found that these cells were depleted even in macroscopically noninflamed colonic areas from patients with UC (Jiang et al., 2025). Experimental depletion increased susceptibility to injury, and an aerolysin-producing *Aeromonas* variant isolated from UC preferentially killed resident macrophages. This work extends the barrier concept beyond epithelial cells and identifies microbial cytotoxicity toward tissue-resident immune cells as a potential initiating event.

Mucus erosion, junctional disorganization, and loss of resident defensive cells are therefore mutually reinforcing defects. Their combined effect is increased access of microbial products to the lamina propria, prolonged inflammatory signaling, and further ecological selection within the lumen.

#### 2.2.3 Immune activation

Dysbiosis and barrier failure promote mucosal immune activation, and the resulting inflammatory environment feeds back on the microbiota. Pattern-recognition receptors (PRRs) expressed by epithelial and immune cells sense microbial and damage-associated molecules (Kawai and Akira, 2009). TLR and NLR signaling can activate NF-κB, MAPK pathways, and inflammasomes, but their effects are context dependent: loss of a receptor may impair protective microbial sensing as well as reduce inflammatory signaling. TLR4-deficient mice, for example, showed altered microbial composition and greater susceptibility to DSS colitis, while human TLR4 expression correlated with *Akkermansia muciniphila* colonization (Liu et al., 2022). NLRP12 deficiency likewise produced spontaneous inflammation, lower diversity, loss of protective commensals, and expansion of inflammation-associated taxa (Chen et al., 2017). These findings caution against treating PRR inhibition as uniformly beneficial.

Mucosal antibodies and antimicrobial factors also shape the ecological boundary. Altered IgA and IgG coating patterns of intestinal bacteria have been associated with disease activity and specific community structures in inflammatory bowel disease (Fang et al., 2021; Gubatan et al., 2021; Masu et al., 2021; Bourgonje et al., 2022). Thapa et al. (2025) identified increased IgA-coated bacterial extracellular vesicles in UC colon; these vesicles triggered proinflammatory responses in CD89-positive immune cells. Antibody coating may therefore function both as a marker of host recognition and as a mechanism that amplifies responses to selected microbial products.

Innate immune cells are direct targets of microbial signals. *Peptostreptococcus anaerobius* promoted macrophage pyroptosis and IL-1β release through TLR2/4–NF-κB–NLRP3 signaling and aggravated DSS colitis (Shen et al., 2024). In contrast, an SCFA-producing bacterial consortium promoted an M2-like macrophage phenotype, and macrophage depletion attenuated its protective effect (Zhao H. et al., 2024). The direction of macrophage regulation thus depends on microbial function and metabolite context rather than on microbial exposure *per se*.

Adaptive immunity is similarly coupled to microbial ecology. In patients responding to fecal microbiota transplantation (FMT), sustained *Faecalibacterium* colonization correlated negatively with Th17 cells and positively with regulatory T cells (Huang C. et al., 2022). In experimental colitis, *F. prausnitzii*, *Bacteroides faecis*, and *R. intestinalis* reduced inflammation while improving the Th17/Treg balance and barrier integrity (Mohebal et al., 2023). These studies support a relationship between commensal restoration and immune rebalancing, although correlation in human FMT studies cannot identify which transferred organism or metabolite is responsible.

The resulting model is circular rather than linear: dysbiosis alters metabolites and microbial effectors; barrier failure increases host exposure; immune activation changes oxygenation, nutrients, and antimicrobial pressure; and these host changes further remodel the microbial community. Therapeutic claims should therefore be evaluated according to where, and with what evidence, an intervention interrupts this cycle (Figure 1).

**FIGURE 1:**
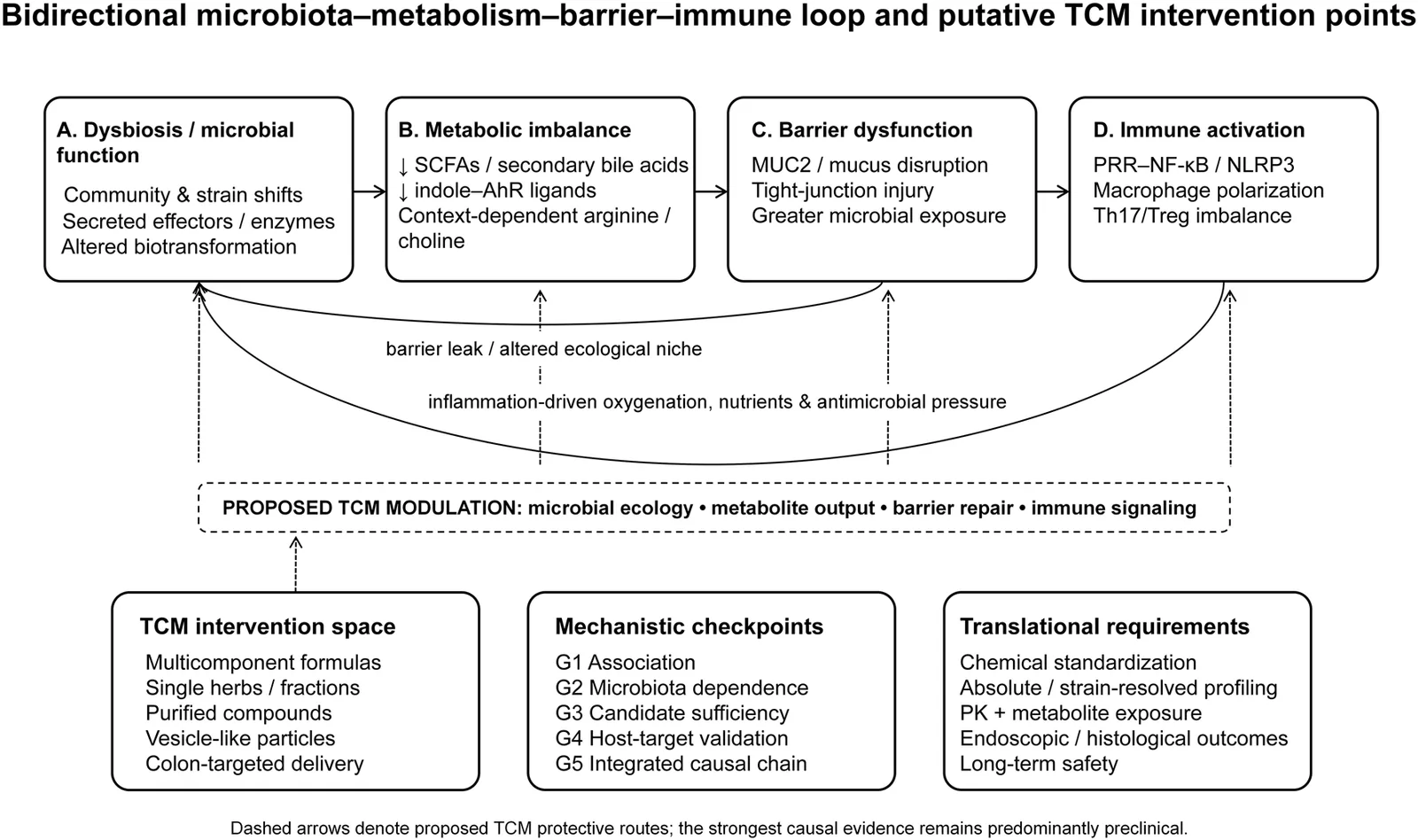
Bidirectional microbiota–metabolism–barrier–immune interactions and proposed TCM intervention points in ulcerative colitis and experimental colitis. Microbial dysbiosis alters metabolites and secreted effectors; metabolic changes weaken mucus and epithelial junctions; barrier failure increases mucosal exposure to microbial products; and inflammation feeds back by changing oxygenation, nutrient availability, antimicrobial pressure, and the ecological niche. Dashed arrows denote putative TCM-mediated protective routes involving multicomponent formulas, single herbs or fractions, purified compounds, vesicle-like particles, and colon-targeted delivery systems. The mechanistic-checkpoint panel outlines five levels: association, microbiota dependence, candidate sufficiency, host-target validation, and integrated causal-chain validation. Most TCM links shown remain predominantly preclinical.

## 3 Traditional Chinese medicine and microbiota modulation in ulcerative colitis

### 3.1 TCM concepts and testable therapeutic hypotheses

#### 3.1.1 Traditional concepts and terminology

Within TCM, UC is commonly discussed under categories such as dysentery (liji), intestinal flux (changpi), and persistent dysenteric discharge (zhixia). Contemporary descriptions often frame spleen deficiency as an underlying susceptibility and damp-heat, toxin, or blood stasis as context-dependent manifestations (Liu et al., 2026; Dong et al., 2025). These labels can guide individualized prescribing within TCM practice, but they should not be treated as validated microbial biomarkers. Inter-rater studies of TCM diagnosis across conditions have generally found low-to-moderate agreement and substantial methodological heterogeneity (Jacobson et al., 2019). For microbiome pharmacology, syndrome differentiation is therefore better used as a prospectively defined clinical stratifier with standardized diagnostic criteria and blinded reproducibility assessment. A claimed syndrome–microbiome biomarker should be replicated across centers, sequencing pipelines, disease phases, and medication strata before it is used for treatment selection.

#### 3.1.2 Therapeutic principles as testable hypotheses

Spleen-tonifying and qi-replenishing interventions are generally used during remission or prolonged disease. Experimental studies have reported treatment-associated shifts in taxa linked in other settings to SCFA production, together with higher acetate or butyrate and improved epithelial or immune endpoints. Combined polysaccharides from *Codonopsis pilosula* and *Astragalus membranaceus* altered community composition and reduced mucosal injury in experimental colitis (Tang et al., 2021). Codonopsis polysaccharide was associated with higher relative abundance of *Ligilactobacillus* and *Akkermansia* and higher acetate and butyrate (Zhou et al., 2025), while *Atractylodes macrocephala* polysaccharide produced other community shifts (Feng et al., 2020). These genus-level relative-abundance changes should not be described as microbiome “improvement” unless they are linked to strain identity, absolute abundance, microbial function, or depletion/reconstitution evidence.

Heat-clearing, dampness-draining, and detoxifying interventions are used when damp-heat and toxin patterns predominate. Baicalin partly corrected community imbalance and limited Proteobacteria expansion in TNBS colitis (Zhu et al., 2020). A lipophilic baicalin derivative altered Lachnospiraceae- and Clostridia-related taxa, reduced reactive oxygen species, and lowered IL-1β, IL-18, and TNF-α (Guo et al., 2025a). Berberine, a major constituent of *Coptis chinensis*, modified *Bacteroides fragilis*-associated communities and suppressed dendritic-cell activation and Th17 differentiation (Zheng et al., 2021). The recurring pharmacological theme is not simply “heat clearing,” but concurrent effects on microbial ecology, redox stress, and inflammatory-cell differentiation.

Qi-regulating, blood-activating, and collateral-unblocking therapy is used for tenesmus and bloody stools attributed to qi stagnation and blood stasis. *Panax notoginseng* saponins altered microbial composition and bile acid profiles and improved the Th17/Treg balance in colitic animals (Wang L. et al., 2025). Safflower polysaccharide increased *Akkermansia* and *Limosilactobacillus*, reduced *Bacteroides*, and increased tight-junction and mucin expression (Qi et al., 2024). These findings provide biological hypotheses for this therapeutic category, but the evidence remains preclinical and should not be read as proof that traditional indications map to a unique microbial signature.

Across therapeutic principles, the reported microbial effects overlap substantially. SCFA recovery, barrier repair, and suppression of inflammatory signaling recur in formulas assigned to different TCM categories. This convergence may reflect shared chemical constituents and common host pathways rather than syndrome-specific microbiome mechanisms. Comparative studies using standardized preparations and prespecified syndrome–microbiome interactions are needed to determine whether the traditional categories predict differential pharmacological effects.

### 3.2 Pharmacological interventions across formulation scales

#### 3.2.1 Multicomponent herbal formulas

Studies of Chinese herbal formulas differ markedly in mechanistic depth. Most combine disease phenotyping with 16S rRNA profiling and report parallel changes in taxa, cytokines, or barrier proteins (G1). Stronger studies test microbiota dependence or transferability with antibiotic depletion or FMT (G2). Defined-strain or metabolite reconstitution can establish partial sufficiency (G3), while receptor antagonism, genetic perturbation, or direct target-engagement is required for host-target validation (G4). Only designs that link microbiota dependence, a manipulated candidate mediator, and a perturbed host target qualify as an integrated causal chain (G5). Organoid responses without target perturbation remain supportive functional evidence rather than target validation.

##### 3.2.1.1 Spleen-tonifying, qi-replenishing, and dampness-draining formulas

Sijunzi Decoction is among the better characterized spleen-tonifying formulas in experimental colitis. In DSS colitis, it reduced tissue injury, increased goblet cells and MUC2 secretion, upregulated ZO-1, occludin, JAM-A, and claudin-1, and reduced claudin-2. Microbiota profiling showed suppression of Proteobacteria and *Escherichia–Shigella* and changes in *Akkermansia muciniphila* and Lachnospiraceae_NK4A136_group (Wu et al., 2023; Li H. et al., 2024). These parallel effects identify a coherent barrier–microbiota phenotype but do not, by themselves, establish mediation.

Mechanistic experiments strengthen the case for microbiota involvement. *Akkermansia* occupies the mucus niche and can influence MUC2 production, while cross-feeding with butyrate-producing taxa may support barrier metabolism (Everard et al., 2013; Belzer et al., 2017; Chia et al., 2018; Zhang H. et al., 2025). Antibiotic depletion weakened the protective effect of Sijunzi Decoction, and FMT from treated donors transferred part of the phenotype (Wu et al., 2023). Metabolomics further identified bile acid remodeling, including increased tauroursodeoxycholic acid. Animal studies of spleen-deficiency models and *in vitro* fermentation provide additional evidence for microbial metabolism and SCFA production (Dong et al., 2024; Gao et al., 2018). Clinical efficacy in UC, however, has not been established by these experiments, and the active chemical constituents responsible for microbiota modulation remain insufficiently resolved.

Shenling Baizhu San has been studied through complementary microbiome, metabolome, and epithelial models. In DSS colitis, the formula partly restored Muribaculaceae and *Ruminococcus* and reduced *Clostridium* expansion (Yu et al., 2022). Metabolomics implicated glycerophospholipid, arachidonic acid, lysine, and transient receptor potential-channel pathways. Drug-containing serum reduced TNF-α-induced oxidative stress, NLRP3 and CASP1 expression, and pyroptosis in intestinal organoids; because this approach does not identify the absorbed compounds reaching the organoid, direct exposure studies with chemically defined constituents would be more informative. For the polysaccharide fraction, antibiotic depletion reduced efficacy and FMT transferred part of the protection (Lv et al., 2022). Recovery of SCFAs, GPR43, kynurenine, AhR, and IL-10 suggests a microbiota–metabolite–host pathway, while a separate study linked the formula to galactose metabolism and bone marrow-associated repair (Li Y. et al., 2024). The evidence supports microbiota dependence more strongly than it supports any single taxon as the mediator.

##### 3.2.1.2 Heat-clearing, dampness-drying, detoxifying, and dysentery-arresting formulas

Huangqin Decoction has been evaluated across progressively stronger designs. Initial studies reported altered diversity and increased *Lactobacillus* in damp-heat-pattern colitis (Zheng et al., 2022). Lactobacilli can acidify the intestinal environment, limit pathogen adhesion, and support mucosal immunoglobulin responses (Fayol-Messaoudi et al., 2005; Mei et al., 2022). A reduction in Chao or Shannon indices should not automatically be interpreted as improvement, because normalization depends on the direction relative to healthy controls and on community function. Subsequent work linked the formula to reduced TLR4/NOD2/RIP2 signaling (Huang S. et al., 2022), suppression of Desulfovibrionaceae-associated epithelial injury, enrichment of a Lachnospiraceae strain, activation of Wnt/β-catenin signaling, and intestinal stem-cell repair (Pan et al., 2026). Metagenomics, targeted metabolomics, antibiotic depletion, and AhR antagonism further showed increased indole derivatives, AhR activation, IL-22 signaling, and Lgr5-positive stem-cell regeneration (Li et al., 2026). Among formula studies, this combination of depletion, strain-level analysis, metabolite measurement, receptor antagonism, and organoid validation provides comparatively strong mechanistic support.

Baitouweng Decoction altered *Escherichia–Shigella*, *Lactobacillus*, and overall community structure in DSS-induced experimental colitis (Chen et al., 2021). Metabolomics linked microbial shifts to a bile acid profile involving TGR5 and FXR signaling (Hua et al., 2021), although correlation alone cannot establish that bile acid remodeling mediates efficacy. Stronger evidence comes from antibiotic depletion, FMT, and indole-3-propionic acid (IPA) supplementation: treated-donor microbiota transferred part of the protection, and IPA supplementation reproduced a defined component of the phenotype while reducing RIPK1/RIPK3/MLKL-dependent epithelial necroptosis (Hu et al., 2025). *Akkermansia muciniphila* supplementation also reproduced part of the FMT-associated phenotype in a damp-heat-pattern model (Zheng et al., 2026b). These experiments support microbiota dependence and partial sufficiency of candidate microbial products or organisms, but they do not by themselves establish necessity or complete mediation by a single taxon/metabolite.

Shaoyao Decoction affected both microbial composition and tryptophan-related metabolism. In DSS colitis, enrichment of Lachnospiraceae_NK4A136_group and *Oscillospira* was accompanied by higher butyrate and lower colonic 5-hydroxytryptamine, with reduced expression of tryptophan hydroxylase 1 and dopa decarboxylase (Zhen et al., 2024). A separate study reported restoration of indole metabolites, AhR activation, IL-22/STAT3 signaling, and epithelial repair (Zhang Y. et al., 2024). Metabolomics also identified region-specific changes in glycosphingolipids, lysophospholipids, and unsaturated fatty acids (Luo et al., 2022). The convergence on tryptophan metabolism is notable, but direct depletion or rescue experiments are still needed to identify the relevant organisms and metabolites.

##### 3.2.1.3 Liver-soothing, spleen-strengthening, and liver–spleen harmonizing formulas

Tongxie Yaofang increased microbial diversity, reduced Proteobacteria, enriched *Akkermansia*-related taxa, and restored acetate and butyrate in experimental colitis (Zheng et al., 2025). FMT from donors treated with Tongxie Yaofang plus Sishen Pill transferred part of the anti-colitis phenotype and linked propionate and butyrate to HIF-1α and HDAC1 regulation (Liu et al., 2025c). Additional studies in diarrhea and irritable bowel syndrome models reported *Lactobacillus johnsonii* colonization and bile salt hydrolase-dependent bile acid signaling (Shao et al., 2024; Jia et al., 2024). These non-UC models are mechanistically suggestive but should not be presented as direct evidence for UC efficacy. The formula therefore has plausible SCFA- and bile acid-related mechanisms, with limited disease-specific causal validation.

##### 3.2.1.4 Cold–heat harmonizing formulas combining elimination and reinforcement

Wumei Pill altered *Allobaculum*, *Bacteroides*, and *Ileibacterium* in chronic colitis and was associated with improved epithelial junctions and immune balance (Wu et al., 2022). In a humanized-microbiota model, modified Wumei Pill increased *Bacteroides thetaiotaomicron* and *B. fragilis* (Zhang C. H. et al., 2025). Independent gnotobiotic work shows that *B. thetaiotaomicron* and *Faecalibacterium prausnitzii* can alter goblet-cell differentiation and mucus glycosylation (Wrzosek et al., 2013), while *B. thetaiotaomicron* can influence AhR-dependent CD4-positive T-cell homeostasis (Li et al., 2021). These external mechanistic studies provide biological context, but they do not prove that the same organisms mediate Wumei Pill efficacy. Direct strain depletion or reconstitution within the formula model is still required.

##### 3.2.1.5 Yang-warming, spleen-strengthening, and astringent antidiarrheal formulas

Lizhong Decoction reduced mucosal injury and shifted Treg, Th17, and Th1 responses in experimental colitis. Antibiotic depletion weakened protection, whereas microbiota transfer from treated donors reproduced part of the effect (Huang et al., 2025). The formula increased *Akkermansia* and Lachnospiraceae_NK4A136_group, reduced *Escherichia–Shigella*, and restored acetate, butyrate, and valerate. Cross-feeding between mucin-utilizing and butyrate-producing organisms provides a plausible ecological explanation (Belzer et al., 2017; Wen et al., 2023). SCFA signaling through FFAR2 and histone deacetylase regulation may contribute to immune effects (Brown et al., 2003; Pan et al., 2018), while metabolomics implicates bile acid, tryptophan, unsaturated fatty acid, and purine pathways (Zou et al., 2020). The dependence experiments support a microbiota-mediated component, but the responsible compounds and microbial functions remain undefined.

Sishen Pill has been examined in models incorporating spleen–kidney yang deficiency as well as conventional DSS injury. Studies have reported increased Firmicutes or *Lactobacillus*, higher butyrate, and improved Treg/Th17 balance (Wang Y. et al., 2022; Li X. et al., 2024). Butyrate can maintain physiological epithelial hypoxia and restrict facultative anaerobic Enterobacteriaceae through PPARγ signaling (Byndloss et al., 2017), and it also affects ERK/STAT3 and ferroptosis-related pathways (Liu et al., 2025d; Chen et al., 2024). A further study linked Sishen Pill to reduced inflammatory dendritic-cell phenotypes (Chen et al., 2020). Most Sishen Pill studies remain associative, however, and syndrome-model procedures introduce additional physiological perturbations that may independently alter the microbiota. Mechanistic claims should therefore be validated in both syndrome-pattern and standard colitis models. Representative TCM formulas evaluated for microbiota-related effects in experimental colitis are summarized in Table 1. Additional pharmacological details for selected multicomponent-formula studies are provided in Supplementary Table S1.

**TABLE 1:** Representative traditional Chinese medicine formulas evaluated for microbiota-related effects in experimental colitis.

Therapeutic principle	Formula	Experimental model	Evidence tier	Principal composition/representative bioactives	Mechanistic design	Microbiota-associated findings	Metabolic and host-response findings
Tonify the spleen and replenish qi	Sijunzi Decoction	3% DSS-induced experimental colitis in mice	G1	*Panax ginseng*, *Atractylodes macrocephala*, *Poria cocos*, *Glycyrrhiza uralensis*; ginsenosides, atractylenolides, polysaccharides, glycyrrhizin	Association: phenotyping and microbiota profiling	↑ *Akkermansia*, Lachnospiraceae_NK4A136_group ↓ *Bacteroides*, *Helicobacter*	↑ ZO-1, occludin, TJP1 ↓ IL-6, IL-1β, TNF-α (Li H. et al., 2024)
Tonify the spleen and replenish qi	Sijunzi Decoction	3% DSS-induced experimental colitis in mice	G2	*Panax ginseng*, *Atractylodes macrocephala*, *Poria cocos*, *Glycyrrhiza uralensis*; ginsenosides, atractylenolides, polysaccharides, glycyrrhizin	Microbiota dependence: antibiotic depletion and FMT	↓ Proteobacteria, *Escherichia-Shigella*	bile acid metabolism ↑ MUC2, ZO-1, occludin, JAM-A, claudin-1/4/11, TUDCA ↓ claudin-2, TNF-α, IL-1β, IFN-γ (Wu et al., 2023)
Tonify the spleen and drain dampness	Shenling Baizhu San	3.5% DSS-induced experimental colitis in mice	G1	Classical multicomponent formula including *Panax ginseng*, *Atractylodes macrocephala*, *Poria cocos*, *Dioscorea opposita*, *Coix lacryma-jobi*, *Lablab album*, *Nelumbo nucifera*, *Platycodon grandiflorus*, *Amomum villosum*, *Glycyrrhiza uralensis*; polysaccharides, ginsenosides, atractylenolides, flavonoids/saponins	Functional association: transcriptomics	↑ *Akkermansia* and Lachnospiraceae_NK4A136_group; ↓ *Escherichia–Shigella* and inflammation-associated members of Ruminococcaceae	↑ ZO-1, occludin, claudin-1 ↓ IL-6, TNF-α (Chen et al., 2026)
Tonify the spleen and drain dampness	Shenling Baizhu San	5% TNBS-induced experimental colitis with a spleen-deficiency and dampness-obstruction pattern in rats; BMSCs	G1	Classical multicomponent formula including *Panax ginseng*, *Atractylodes macrocephala*, *Poria cocos*, *Dioscorea opposita*, *Coix lacryma-jobi*, *Lablab album*, *Nelumbo nucifera*, *Platycodon grandiflorus*, *Amomum villosum*, *Glycyrrhiza uralensis*; polysaccharides, ginsenosides, atractylenolides, flavonoids/saponins	Functional association: metabolomics; BMSC intervention	↑ Lactobacillaceae ↓ Aeromonadaceae	galactose metabolism ↑ occludin, ZO-1, GALK2 ↓ SDF-1, CXCR4, β-galactose, GALM, GLB1, p16, p53, p21, p27 (Li Y. et al., 2024)
Tonify the spleen and drain dampness	Shenling Baizhu San	3% DSS-induced experimental colitis in mice	G2/G4	Classical multicomponent formula including *Panax ginseng*, *Atractylodes macrocephala*, *Poria cocos*, *Dioscorea opposita*, *Coix lacryma-jobi*, *Lablab album*, *Nelumbo nucifera*, *Platycodon grandiflorus*, *Amomum villosum*, *Glycyrrhiza uralensis*; polysaccharides, ginsenosides, atractylenolides, flavonoids/saponins	Dependence and target validation: antibiotic depletion, microbiota transfer, metabolomics, and AhR antagonism	↑ *Ruminococcus*, *Odoribacter*, *Butyricimonas*, *Bifidobacterium*, *Allobaculum*, *Bacteroides*	tryptophan metabolism ↑ IL-10, acetate, propionate, butyrate, GPR43, AhR, CYP1A1 ↓ TNF-α, IL-1β, IFN-γ (Lv et al., 2022)
Tonify the spleen and drain dampness	Shenling Baizhu San	2% DSS-induced experimental colitis in mice	G1*	Classical multicomponent formula including *Panax ginseng*, *Atractylodes macrocephala*, *Poria cocos*, *Dioscorea opposita*, *Coix lacryma-jobi*, *Lablab album*, *Nelumbo nucifera*, *Platycodon grandiflorus*, *Amomum villosum*, *Glycyrrhiza uralensis*; polysaccharides, ginsenosides, atractylenolides, flavonoids/saponins	Functional association and supportive host-functional assay: metabolomics and intestinal organoids	↑ Bacteroidetes, Fusobacteria, *Ruminococcus* ↓ *Clostridium*	Inflammatory regulation through TRP channels; arachidonic acid, glycerophospholipid, and lysine metabolism; ↓ fecal albumin, IL-17, CXCL1, ROS, NLRP3, and CASP1 (Yu et al., 2022)
Tonify the spleen and drain dampness	Shenling Baizhu San	5% TNBS-induced experimental colitis in rats	G1	Classical multicomponent formula including *Panax ginseng*, *Atractylodes macrocephala*, *Poria cocos*, *Dioscorea opposita*, *Coix lacryma-jobi*, *Lablab album*, *Nelumbo nucifera*, *Platycodon grandiflorus*, *Amomum villosum*, *Glycyrrhiza uralensis*; polysaccharides, ginsenosides, atractylenolides, flavonoids/saponins	Association: phenotyping and microbiota profiling	↑ *Prevotella* and *Oscillospira*; ↓ *Desulfovibrio*, *Bilophila*, and *Bacteroides*	↑ SOD and CAT activities; ↓ MPO (Gu et al., 2021)
Clear heat and dry dampness; detoxify and arrest dysentery	Huangqin Decoction	5% DSS-induced damp-heat-pattern experimental colitis in rats	G1	*Scutellaria baicalensis*, *Paeonia lactiflora*, *Glycyrrhiza uralensis*, *Ziziphus jujuba*; baicalin/baicalein, paeoniflorin, glycyrrhizin	Association: phenotyping and microbiota profiling	↑ Firmicutes, *Lactobacillus* ↓ Bacteroidetes, *Treponema*	↓ IL-6, IL-17, IL-23, mtDNA, TLR4, p-PI3K, p-Akt (Zheng et al., 2022)
Clear heat and dry dampness; detoxify and arrest dysentery	Huangqin Decoction	3% DSS-induced experimental colitis in mice	G1	*Scutellaria baicalensis*, *Paeonia lactiflora*, *Glycyrrhiza uralensis*, *Ziziphus jujuba*; baicalin/baicalein, paeoniflorin, glycyrrhizin	Association: phenotyping and microbiota profiling	↓ Deferribacteres, Deferribacteraceae, Alcaligenaceae	↓ RegIIIγ, NOD2, RIP2, NF-κB p65, IL-1β, IL-17, MCP-1, TNF-α, IFN-γ, IL-6 (Huang S. et al., 2022)
Clear heat and dry dampness; detoxify and arrest dysentery	Huangqin Decoction	3.5% DSS-induced experimental colitis in mice	G2/G4	*Scutellaria baicalensis*, *Paeonia lactiflora*, *Glycyrrhiza uralensis*, *Ziziphus jujuba*; baicalin/baicalein, paeoniflorin, glycyrrhizin	Dependence and target validation: antibiotic depletion, metagenomics, targeted metabolomics, and AhR antagonism	↑ Bacteroidota, Muribaculaceae, *Heminiphilus*, *Sangeribacter*, *Lactobacillus* ↓ Bacillota	tryptophan metabolism ↑ ZO-1, E-cadherin, MUC2, IPA, TRM, IAM, AhR, CYP1A1, IL-22 ↓ IL-6, IL-1β, FITC-dextran, 3IS (Li et al., 2026)
Clear heat and dry dampness; detoxify and arrest dysentery	Huangqin Decoction	3% DSS-induced experimental colitis in mice	G1	*Scutellaria baicalensis*, *Paeonia lactiflora*, *Glycyrrhiza uralensis*, *Ziziphus jujuba*; baicalin/baicalein, paeoniflorin, glycyrrhizin	Association: phenotyping and microbiota profiling	↑ Bacteroidetes, *Lactobacillus*, Prevotellaceae, *Blautia* ↓ Firmicutes, Proteobacteria, Epsilonproteobacteri, Deltaproteobacteria, *Helicobacter*	↓ LPS, TLR4, GPCR, RasGTP, PI3K, p-Akt, HIF-1α, p-IKKβ, p-p65, iNOS, CXCL10, IL-1β, IL-6 (Li et al., 2020)
Clear heat and dry dampness; detoxify and arrest dysentery	Huangqin Decoction	2.5% DSS-induced experimental colitis in mice	G2/G3*	*Scutellaria baicalensis*, *Paeonia lactiflora*, *Glycyrrhiza uralensis*, *Ziziphus jujuba*; baicalin/baicalein, paeoniflorin, glycyrrhizin	Microbiota dependence and candidate sufficiency: antibiotic depletion, strain reconstitution, metagenomics, and organoid co-culture	↑ Lachnospiraceae bacterium A2 ↓ Desulfovibrionaceae bacterium	↑ ZO-1, E-cadherin, claudin-1, MUC2, β-catenin/Wnt3a ↓ GM-CSF, IL-1β, IL-6, TNF-α (Pan et al., 2026)
Clear heat and dry dampness; detoxify and arrest dysentery	Baitouweng Decoction	3% DSS-induced experimental colitis in mice	G1	*Pulsatilla chinensis*, *Coptis chinensis*, *Phellodendron chinense*, *Fraxinus rhynchophylla*; triterpenoid saponins, berberine, phellodendrine, aesculin/aesculetin	Association: phenotyping and microbiota profiling	↑ Bacteroidetes, *Lactobacillus*, Verrucomicrobia, *Akkermansia* ↓ Firmicutes, Proteobacteria, *Escherichia-Shigella*	↓ IL-6, IL-1β, TNF-α, p-STAT3/STAT3 (Chen et al., 2021)
Clear heat and dry dampness; detoxify and arrest dysentery	Baitouweng Decoction	3.5% DSS-induced experimental colitis in mice	G1	*Pulsatilla chinensis*, *Coptis chinensis*, *Phellodendron chinense*, *Fraxinus rhynchophylla*; triterpenoid saponins, berberine, phellodendrine, aesculin/aesculetin	Functional association: metabolomics	↑ *Lactobacillus*, *Desulfovibrio*, *Ruminococcus*, and selected Firmicutes; ↓ *Bacteroides*, *Oscillospira*, and *Prevotella*	bile acid metabolism ↑ DCA, TUDCA, THDCA, TCDCA, TDCA, FXR, TGR5 ↓ TNF-α, TGF-β1, TBA, T-CHO, UDCA, HDCA, αMCA, βMCA, CA, GLCA, TβMCA (Hua et al., 2021)
Clear heat and dry dampness; detoxify and arrest dysentery	Baitouweng Decoction	3.5% DSS-induced experimental colitis in mice	G2/G3	*Pulsatilla chinensis*, *Coptis chinensis*, *Phellodendron chinense*, *Fraxinus rhynchophylla*; triterpenoid saponins, berberine, phellodendrine, aesculin/aesculetin	Microbiota dependence/transferability and candidate metabolite sufficiency: antibiotic depletion, FMT, metabolomics, and IPA rescue	↓ Proteobacteria, Enterobacteriaceae	tryptophan metabolism ↑ MUC2, ZO-1, claudin-4, occludin, IPA ↓ MPO, IL-6, TNF-α, IL-1β, CXCL1, FITC-dextran, p-RIPK1, p-RIPK3, p-MLKL (Hu et al., 2025)
Clear heat and dry dampness; detoxify and arrest dysentery	Baitouweng Decoction	3% DSS-induced large-intestinal damp-heat-pattern experimental colitis in mice	G2	*Pulsatilla chinensis*, *Coptis chinensis*, *Phellodendron chinense*, *Fraxinus rhynchophylla*; triterpenoid saponins, berberine, phellodendrine, aesculin/aesculetin	Transferability: FMT	↑ *Akkermansia muciniphila*	↑ IL-10, IL-4, ZO-1, occludin, MUC2, claudin-1, Foxp3 ↓ TNF-α, IL-1β, IL-6, RORγt, CaSR, Gnaq, Gna11, MAPK (Zheng et al., 2026b)
Clear heat and dry dampness; detoxify and arrest dysentery	Shaoyao Decoction	5% DSS-induced experimental colitis in mice	G1	*Paeonia lactiflora*-centered multicomponent formula with *Coptis*, *Scutellaria*, *Angelica*, *Aucklandia*, *Rheum*, *Glycyrrhiza* and related ingredients; paeoniflorin, berberine, baicalin, anthraquinones, ferulic-acid-related constituents	Functional association: transcriptomics and metabolomics	↑Lachnospiraceae_NK4A136_group, *Oscillospira*	SCFA, tryptophan, and 5-HT metabolism; ↑ MAOB, ALDH1B1, SLC6A4, and butyrate; ↓ 5-HT, Tph1, Ddc, IL-1β, IL-2, IL-6, TNF-α, and IFN-γ (Zhen et al., 2024)
Clear heat and dry dampness; detoxify and arrest dysentery	Shaoyao Decoction	3.5% DSS-induced damp-heat-pattern experimental colitis in mice	G1	*Paeonia lactiflora*-centered multicomponent formula with *Coptis*, *Scutellaria*, *Angelica*, *Aucklandia*, *Rheum*, *Glycyrrhiza* and related ingredients; paeoniflorin, berberine, baicalin, anthraquinones, ferulic-acid-related constituents	Functional association: metabolomics	↑ Bacteroidetes, *Lactobacillus*, *Akkermansia*, Verrucomicrobia ↓ Proteobacteria, *Desulfovibrio*, *Helicobacter*, *Escherichia*, *Acetatifactor*	tryptophan metabolism ↑ IL-10, T-AOC, IA, IAA, IAld, IGA, IPA, AhR, CYP1A1, IL-22, Reg3β, Reg3γ, ZO-1, occludin ↓ IL-6, IL-1β, TNF-α, MDA (Zhang Y. et al., 2024)
Soothe the liver and strengthen the spleen; harmonize the liver and spleen	Tongxie Yaofang	5% DSS-induced experimental colitis in mice	G1	*Atractylodes macrocephala*, *Paeonia lactiflora*, *Citrus reticulata*, *Saposhnikovia divaricata*; atractylenolides, paeoniflorin, hesperidin/nobiletin, chromones	Functional association: metabolomics	↑ Verrucomicrobia, Verrucomicrobiaceae, *Akkermansia*, *A. muciniphila*, *Ruminococcus*, TM7, F/B ↓ Proteobacteria, Desulfovibrionaceae, *Desulfovibrio*	SCFA metabolism ↑ acetate, butyrate, IL-10 ↓ propionate, IL-6, TNF-α (Zheng et al., 2025)
Harmonize cold and heat; combine elimination and reinforcement	Wumei Pill	2% DSS-induced experimental colitis in mice	G1	Classical *Prunus mume*-centered multicomponent formula with *Coptis*, *Phellodendron*, *Panax ginseng*, *Angelica*, *Zingiber*, *Asarum* and *Cinnamomum*-related ingredients; organic acids, berberine, ginsenosides, volatile constituents	Association: phenotyping and microbiota profiling	↑ *Allobaculum*, *Bacteroides* ↓ *Ileibacterium*	↑ E-cadherin, occludin, ZO-1, claudin-4, claudin-5, claudin-8 ↓ claudin-1, IFN-γ, RORγt, IL-17A, TNF-α, IL-1β (Wu et al., 2022)
Harmonize cold and heat; combine elimination and reinforcement	Wumei Pill	2% DSS-induced experimental colitis in mice	G1	Classical *Prunus mume*-centered multicomponent formula with *Coptis*, *Phellodendron*, *Panax ginseng*, *Angelica*, *Zingiber*, *Asarum* and *Cinnamomum*-related ingredients; organic acids, berberine, ginsenosides, volatile constituents	Association: phenotyping and microbiota profiling	↑ Bacteroidota, Proteobacteria, Muribaculaceae, *Escherichia-Shigella* ↓ Firmicutes, Actinobacteriota, Clostridia_UCG-014	↑ IL-10 ↓ IL-6, TNF-α, p-p65 (Zhao G. Y. et al., 2024)
Harmonize cold and heat; combine elimination and reinforcement	Modified Wumei Pill	Humanized-microbiota mouse model of experimental colitis with a damp-heat and blood-stasis pattern	G1†	Modified formula; study-specific composition differs from the classical Wumei Pill and should be interpreted separately from classical-preparation studies.	Contextual validation: humanized-microbiota model	↑ Firmicutes, Proteobacteria, Fusobacteriota, Actinobacteriota, Gemmatimonadota, *Bacteroides thetaiotaomicron*, *B. fragilis*, *B. ovatus*	↓ Th17, Th17/Treg (Zhang C. H. et al., 2025)
Warm yang and strengthen the spleen; astringe the intestine and relieve diarrhea	Lizhong Decoction	3% DSS-induced experimental colitis in mice	G2	*Panax ginseng*, *Atractylodes macrocephala*, dried *Zingiber officinale*, *Glycyrrhiza uralensis*; ginsenosides, atractylenolides, gingerols/shogaols, glycyrrhizin	Microbiota dependence: antibiotic depletion, FMT, and metabolomics	↑ Lachnospiraceae_NK4A136_group, *Akkermansia* ↓ *Escherichia-Shigella*	SCFA metabolism ↑ acetate, butyrate, valerate, Treg, FFAR2, claudin-1 ↓ Th17, Th1, HDAC6 (Huang et al., 2025)
Warm yang and strengthen the spleen; astringe the intestine and relieve diarrhea	Lizhong Decoction	Sequential 2% and 2.5% DSS-induced experimental colitis in mice	G1	*Panax ginseng*, *Atractylodes macrocephala*, dried *Zingiber officinale*, *Glycyrrhiza uralensis*; ginsenosides, atractylenolides, gingerols/shogaols, glycyrrhizin	Functional association: metabolomics	↑ Bacteroidetes, *Blautia*, unclassified Muribaculaceae, Prevotellaceae UCG-001, *Ruminiclostridium* 9 ↓ Proteobacteria, Gammaproteobacteria, *Escherichia-Shigella*, *Clostridium* sensu stricto 1, *Enterobacter*	Bile acid, tryptophan, unsaturated fatty acid, and glycerophospholipid metabolism; ↑ cholic acid, α-linolenic acid, stearidonic acid, and L-tryptophan; ↓ adenosine, lysoPC(18:0), glycocholic acid, and deoxycholic acid (Zou et al., 2020)
Warm yang and strengthen the spleen; astringe the intestine and relieve diarrhea	Sishen Pill	Senna leaf, hydrocortisone, and DNBS/ethanol-induced experimental colitis with spleen–kidney yang deficiency in rats	G1	*Psoralea corylifolia*, *Myristica fragrans*, *Schisandra chinensis*, *Tetradium ruticarpum* with traditional adjuncts; psoralen/isopsoralen, lignans, evodiamine/rutaecarpine, volatile constituents	Functional association: metabolomics	↑ Firmicutes, *Lactobacillus* ↓ Bacteroidota	SCFA metabolism ↑ butyrate, TGF-β1, PPARγ, Treg, IL-10 ↓ STAT3/p-STAT3, Th17, IL-6, IL-17 (Wang Y. et al., 2022)
Warm yang and strengthen the spleen; astringe the intestine and relieve diarrhea	Sishen Pill	Rhubarb, hydrocortisone, and 3% DSS-induced experimental colitis with spleen–kidney yang deficiency in mice	G1	*Psoralea corylifolia*, *Myristica fragrans*, *Schisandra chinensis*, *Tetradium ruticarpum* with traditional adjuncts; psoralen/isopsoralen, lignans, evodiamine/rutaecarpine, volatile constituents	Functional association: metabolomics	↑ unclassified Lachnospiraceae, Lachnospiraceae_UCG-006, *Parvibacter*, *Akkermansia*, *Rhodococcus*, Verrucomicrobiota, Akkermansiaceae ↓ *Aerococcus*	↑ IL-10, IκB ↓ TNF-α, IL-1β, IL-6, IL-12p70, TLR4, MyD88, TRAF6, TAB2, NF-κB p65 (Ge et al., 2022)
Warm yang and strengthen the spleen; astringe the intestine and relieve diarrhea	Sishen Pill	3% DSS-induced experimental colitis in mice	G1	*Psoralea corylifolia*, *Myristica fragrans*, *Schisandra chinensis*, *Tetradium ruticarpum* with traditional adjuncts; psoralen/isopsoralen, lignans, evodiamine/rutaecarpine, volatile constituents	Association: phenotyping and microbiota profiling	↑ *Akkermansia*, *Corynebacterium* ↓ Lachnospiraceae_NK4A136_group	↓ IL-1β, IL-4, IL-9, IL-17A, CD11c+CD103+E-cadherin+ cells, CD11c+CD103+TNF-α+ cells (Chen et al., 2020)

Abbreviation and evidence key: AhR, aryl hydrocarbon receptor; DNBS, 2,4-dinitrobenzenesulfonic acid; DSS, dextran sulfate sodium; FMT, fecal microbiota transplantation; SCFA, short-chain fatty acid; TNBS, 2,4,6-trinitrobenzenesulfonic acid; G1, association; G2, microbiota dependence/transferability; G3, candidate sufficiency; G4, host-target validation; G5, integrated causal chain. Combined labels (for example, G2/G4) indicate that a study satisfies more than one nonsequential criterion without meeting the complete G5 chain. *Organoid or cell assays without target perturbation are supportive functional evidence and do not independently qualify as G4. †Humanized-microbiota context alone does not establish mediation. Upward/downward arrows denote changes relative to disease-model controls. Relative-abundance shifts do not establish microbial benefit or causality, and rows should not be interpreted as efficacy rankings.

#### 3.2.2 Single herbs, functional fractions, and purified constituents

##### 3.2.2.1 Extracts and functional fractions from single herbs

Single-herb extracts and functional fractions offer a narrower chemical space than multicomponent formulas and can therefore help identify the material basis of microbiota modulation. The preparations studied include aqueous and alcoholic extracts, polysaccharides, fructans, volatile oils, flavonoid fractions, alkaloids, and phenolic acids. Polysaccharides and fructans are particularly relevant because they can reach the colon and function as fermentation substrates, whereas smaller absorbed constituents may act directly on host pathways before or after microbial biotransformation. Distinguishing these exposure routes is essential for pharmacological interpretation.

Indigo naturalis, *Glycyrrhiza uralensis*, and *Poria cocos* illustrate the common pattern of parallel microbial, barrier, and inflammatory changes. Indigo naturalis increased *Lactobacillus*, enhanced mucus production, and suppressed TLR4/MyD88/NF-κB signaling in DSS colitis (Yang Q. Y. et al., 2021).

*Glycyrrhiza uralensis* extract increased *Akkermansia muciniphila*, reduced TLR4/MyD88/NF-κB signaling, and improved barrier and T-helper-cell endpoints (Shi et al., 2022). *Poria cocos* extract restored ZO-1 and claudin-1 while reducing NF-κB-associated inflammation and altering the microbiota (Song et al., 2024). These studies show coordinated pharmacology but do not identify a necessary organism or microbial product. Without depletion or rescue experiments, the microbial changes should be described as treatment-associated rather than as confirmed mediators.

*Aucklandia lappa* and *Codonopsis pilosula* have been examined with more explicit microbe–metabolite–host designs. *Aucklandia lappa* enriched *Lactobacillus reuteri* and increased indole-3-carboxaldehyde, an AhR ligand that inhibited inflammatory macrophage polarization; antibiotic depletion, strain supplementation, and metabolite supplementation supported partial microbiota dependence (Tang et al., 2026). *Codonopsis pilosula* reduced *Escherichia*–Shigella, increased *Lactobacillus* and *Alistipes*, and restored 5-hydroxyindoleacetic acid and arachidonic acid metabolites (Yang et al., 2026). AhR activation, reduced NF-κB p65 and COX-2, and loss of efficacy after antibiotic depletion support a linked tryptophan–AhR and arachidonic acid mechanism, although the contribution of direct host exposure to Codonopsis constituents remains to be quantified.

*Portulaca oleracea*, *Origanum vulgare*, and Xuesanqi each produced broad ecological and metabolic changes. A polyphenol-rich Portulaca extract restored barrier proteins, altered carbohydrate metabolism, and inhibited NLRP3/NF-κB signaling (Li Z. et al., 2025). Oregano extract modified *Escherichia–Shigella*, Bacteroidetes, bile acids, and SCFAs (Yu et al., 2023). Xuesanqi increased *Lactobacillus*, Muribaculaceae, and *Akkermansia*, raised acetate and propionate, activated GPR41/GPR43/GPR109A, and reduced MAPK signaling (Su et al., 2024). These multi-endpoint studies are useful for hypothesis generation, but their breadth can also make causal attribution difficult.

Polysaccharides and fructans are the most extensively studied functional fractions. Codonopsis polysaccharide increased *Ligilactobacillus*, *Akkermansia*, *Faecalibaculum*, and *Odoribacter*, raised acetate and butyrate, and affected the GPR43/GPR109A–NLRP3 axis; FMT transferred part of the protection (Zhou et al., 2025). Ginseng polysaccharides altered *Lactobacillus* and tryptophan metabolites and improved barrier function through 5-HT/HTR3A signaling; antibiotic depletion and FMT again supported microbiota dependence (Wan et al., 2024). *Atractylodes macrocephala* polysaccharide restored Muribaculaceae and *Akkermansia* and was linked to JAK/STAT/IRF1-related epithelial immunity (Kong et al., 2025). Polygonatum fractions showed partly distinct mechanisms: steamed *Polygonatum sibiricum* polysaccharides restored Muribaculaceae and SCFAs (Li H. et al., 2025), whereas a low-molecular-weight *Polygonatum cyrtonema* fructan acted through the microbiota and directly targeted follistatin/BMP4/Smad1/ID1 signaling (Xu et al., 2026). These examples demonstrate that a fraction can combine prebiotic-like effects with direct host pharmacology.

Alkaloid, phenolic acid, and volatile-oil fractions provide a complementary view. *Corydalis saxicola* alkaloids enriched *Lactobacillus johnsonii* and altered linoleic acid metabolism (Jin et al., 2025). Chicory-root phenolic acids modified the microbiota and reduced TRPV4/TRPA1–NLRP3/GSDMD signaling (Tian et al., 2024), while *Angelica sinensis* oil affected sphingolipid, arachidonic acid, and glycerophospholipid pathways (Liu C. et al., 2024). Fractionation narrows the chemical range but does not eliminate complexity. Batch composition, molecular-weight distribution, extraction conditions, and contaminating small molecules must be characterized before a specific microbiota mechanism can be assigned.

##### 3.2.2.2 Purified bioactive compounds from Chinese herbs

Purified compounds have defined structures and are better suited than crude extracts to pharmacokinetic analysis, target validation, and dose–response testing. Even so, a compound-induced microbial shift may result from direct antibacterial activity, altered host secretion, changes in intestinal transit, or microbial biotransformation. Mechanistic studies should therefore separate direct host targets from microbiota-dependent effects rather than treating them as mutually exclusive explanations.

Several compounds converge on microbial metabolites and AhR/NLRP3 signaling. Anemoside B4 increased butyrate and propionate, activated AhR, reduced oxidative stress, and inhibited the NLRP3 inflammasome while improving epithelial junctions (Wu et al., 2025). Gastrodin restored Muribaculaceae and *Lactobacillus*, increased tryptophan metabolites, and suppressed NLRP3/ASC/caspase-1/IL-1β signaling through AhR (Zhang D. et al., 2025). Paeonol increased *Clostridium butyricum* and SCFAs and enhanced the ILC3/IL-22 response; loss of efficacy in pseudo-germ-free mice and recovery after C. butyricum supplementation support mediation by a defined organism (Zhao et al., 2023). Astragaloside IV restored butyrate-related taxa, increased butyrate, and improved the Th17/Treg balance (Zhong et al., 2026). The paeonol study is mechanistically stronger because it includes both depletion and strain rescue.

Other purified compounds have been linked to specific metabolites or host signaling nodes. Asiatic acid promoted microbiota-dependent production of γ-glutamyltyrosine, activated cAMP/protein kinase A signaling, and inhibited NF-κB (Zheng et al., 2026a). Ginsenoside compound K improved permeability and restored occludin, ZO-1, E-cadherin, and MUC2 while reducing *Bacteroides* and *Romboutsia* and increasing *Akkermansia*; microbiota transfer reproduced part of the protection (Wang L. et al., 2022). These studies move beyond generic anti-inflammatory descriptions, but quantitative exposure data are still needed to determine whether the relevant concentrations are achieved in the lumen, mucosa, or systemic circulation.

Formononetin lost much of its effect after microbiota depletion and regained activity after FMT, together with correction of macrophage polarization (Xiao et al., 2024). Dehydroevodiamine altered microbial and serum metabolite profiles while directly targeting AKT1 and suppressing PI3K/AKT/NF-κB signaling (Ma et al., 2024). Baicalein affected the Nrf2/System Xc−/GPX4/ACSL4 ferroptosis axis and microbial structure (Lv et al., 2025). Matrine, morin, ophiopogonin D, obacunone, pinocembrin, and capilliposide A have also been associated with microbiota remodeling, barrier protection, and reduced inflammation (Mao et al., 2024; Qiu et al., 2024; Zhang T. et al., 2024; Luo et al., 2020; Yue et al., 2020; Zhao et al., 2025). For most of these compounds, 16S profiling and correlation remain the principal microbial evidence; strain-level, metabolite-rescue, and target-engagement studies are still uncommon.

#### 3.2.3 Vesicle-like particles, engineered delivery, and processing

Plant-derived extracellular vesicle-like particles are being investigated as both bioactive cargo and delivery vehicles. *Atractylodes macrocephala*-derived particles altered microbial diversity, tryptophan metabolism, indole production, barrier injury, and Th17-related pathways (Tan et al., 2025). Flos Sophorae Immaturus-derived nanovesicles enriched *Lactobacillus*, increased indole-3-acetic acid, activated AhR, and inhibited NLRP3 and oxidative stress (Wu et al., 2026). Antibiotic depletion, FMT, metabolite supplementation, AhR antagonism, and intestinal organoids provided a comparatively complete causal chain. Reproducible isolation, cargo characterization, dose normalization, stability, and distinction from non-vesicular contaminants are now essential for translation.

Honeysuckle-derived MIR2911 suggests a different form of cross-kingdom regulation. Orally administered plant small RNA was reported to enter host extracellular vesicles and reach intestinal bacteria, where it suppressed the replication gene dnaG, reduced *Escherichia–Shigella* expansion, and ameliorated colitis (Li W. et al., 2024). The absence of the same effect with free MIR2911 supports a delivery-dependent mechanism. Independent replication and rigorous controls for sequence specificity, vesicle loading, and RNA stability will be important because dietary small-RNA transfer remains technically challenging.

Engineered formulations can increase colonic exposure and may also change which microbial communities encounter an herbal payload. pH-responsive colon-targeted microspheres containing phosphorylated wild-ginseng polysaccharides improved colonic retention, reduced TLR4/MyD88 signaling, shifted macrophage phenotypes, and altered microbial/metabolic profiles in experimental colitis (Huo et al., 2025). A rhubarb-polysaccharide–berberine hydrogel prolonged residence at inflamed sites and was associated with *Akkermansia*/Muribaculaceae and bile-acid changes (Guo et al., 2025b). These delivery systems create additional pharmacological variables that should be reported explicitly: payload loading, particle size, release kinetics across intestinal pH, mucosal residence, carrier degradation, local versus systemic exposure, and batch reproducibility. Carrier-only and free-drug controls are essential because the biomaterial itself can alter mucus, transit, immune sensing, or microbial growth. Translation will also require stability studies during storage and gastrointestinal passage, scalable manufacturing, and toxicology of both carrier and cargo.

Processing and herbal compatibility can also alter microecological pharmacology. Vinegar-processed frankincense showed greater activity than raw material and was linked to bile salt hydrolase-active *Bifidobacterium longum* and *Lactobacillus acidophilus* and to altered glycodeoxycholic acid hydrolysis (Lu et al., 2025). Bran-fried *Atractylodes lancea* polysaccharides produced stronger microbial, metabolic, and barrier effects than unprocessed material (Yao et al., 2025). Berberine hydrochloride and dehydrocostus lactone displayed partly complementary effects on *Akkermansia*, diversity, barrier proteins, and SCFAs, and FMT transferred part of the combined phenotype (Xu et al., 2023). These observations support processing and compatibility as pharmacologically meaningful variables, but chemical fingerprinting is required to link processing-induced compositional changes to microbial function.

### 3.3 Strength of evidence and causal attribution

Across intervention classes, G1 association remains the dominant level of evidence. A treatment-associated rise in *Lactobacillus*, *Akkermansia*, or another taxon commonly considered favorable is not, by itself, evidence of beneficial microbial activity. Relative abundance can increase because competing organisms decline, and microbial shifts may be secondary to lower inflammation, altered food intake, transit, host secretion, or direct antimicrobial exposure. More persuasive inference requires absolute quantification, strain-level identification, functional or metabolite measurements, and experiments that manipulate the candidate organism or product.

Antibiotic depletion and fecal microbiota transplantation (FMT) strengthen inference but are not definitive. Antibiotics can alter host immunity and epithelial biology independently of microbial depletion, whereas FMT transfers metabolites, bacteriophages, fungi, and host material together with bacteria. Direct herbal activity should therefore be separated from microbiota-dependent activity with matched-exposure designs: compare conventional and microbiota-depleted animals; test whether treated-donor microbiota transfers a phenotype; quantify luminal and systemic exposure to parent compounds and microbial metabolites; use defined-strain or metabolite reconstitution; and validate the relevant host target with antagonists or genetic perturbation. Cell or organoid assays should use concentrations anchored to measured exposure and include carrier-only controls for engineered delivery systems. Loss of efficacy after microbial depletion plus recovery by a defined microbial product provides stronger evidence than either experiment alone.

Table 2 summarizes the conclusions that can—and cannot—be drawn from commonly used experimental designs. This evidence hierarchy should guide interpretation of the current literature and the design of future TCM–microbiome studies.

**TABLE 2:** Revised evidence hierarchy for microbiota-related pharmacology in TCM and comparator studies of ulcerative colitis/experimental colitis.

Evidence grade	Typical design	Inference supported	Limitations and representative examples
G1 Association	16S/shotgun profiling with histology, cytokines, barrier proteins, or untargeted metabolomics	Microbial and host changes co-occur after treatment	Reverse causation; relative-abundance bias; model/batch effects. Examples: (Li H. et al., 2024; Yu et al., 2022; Gu et al., 2021).
G2 Microbiota dependence/transferability	Antibiotic depletion, germ-reduced conditions, or FMT	The microbiota contributes to efficacy or transfers part of the phenotype	Antibiotic off-target effects; FMT transfers metabolites, fungi, phages, and host material. Examples: (Wu et al., 2023; Huang et al., 2025; Xiao et al., 2024).
G3 Candidate sufficiency	Defined-strain or metabolite supplementation/reconstitution	A candidate organism or metabolite is sufficient for part of the phenotype	Sufficiency does not prove necessity or mediation; exposure may be nonphysiological. Examples: (Pan et al., 2026; Hu et al., 2025; Tang et al., 2026; Zhao et al., 2023).
Supportive host-functional assay (not a causal grade)	Organoids, epithelial cells, or co-culture without receptor/genetic perturbation	Shows a host response under controlled exposure	Does not by itself validate a target or microbiota-mediated mechanism. Examples: (Yu et al., 2022; Pan et al., 2026).
G4 Host-target validation	Receptor antagonism/knockout, genetic perturbation, or direct target-engagement	Validates the receiving host pathway for a microbial or herbal signal	Target relevance remains model- and exposure-dependent. Examples: (Liu et al., 2023; Li et al., 2026; Xu et al., 2026; Ma et al., 2024).
G5 Integrated causal chain	Microbiota-dependence test + defined candidate manipulation + host-target perturbation, ideally with necessity and sufficiency	Links intervention → microbiota/function → mediator → host target → phenotype	Highest mechanistic confidence, but still requires replication and pharmacokinetic plausibility. Example: (Wu et al., 2026).
Clinical translation benchmark	Randomized human trials with objective clinical/endoscopic outcomes; ideally paired microbiome/metabolome sampling	Tests human efficacy and biomarker performance	Clinical efficacy does not itself prove microbiota mediation. Reference (Firoozi et al., 2024) is sodium butyrate, a microbiota-derived metabolite benchmark rather than TCM evidence; human TCM/botanical trials are summarized in Table 3.

## 4 Discussion and translational perspectives

### 4.1 From taxonomic shifts to pharmacological mechanisms

The literature supports a plausible bidirectional pharmacological model. Herbal constituents can alter nutrient availability, microbial growth, virulence, and metabolite production; the microbiota can in turn transform glycosides, alkaloids, polysaccharides, and other constituents into products with different absorption and target profiles. Recent syntheses and a representative postbiotic study likewise emphasize that microbial function, barrier repair, and immune regulation are coupled rather than separable endpoints (Wang et al., 2024; Zhou et al., 2024; Han et al., 2025; Zhang T. et al., 2025; Chen et al., 2025). Yet most UC studies measure only one side of this interaction. Relative-abundance data are often interpreted as mechanism without absolute microbial load, strain resolution, microbial gene expression, metabolite flux, or compound pharmacokinetics. The field should therefore move from “which taxa changed?” to “which microbial function changed, which herbal constituent caused it, and which host target received the resulting signal?”

### 4.2 Methodological limitations, bias, and causal inference

Animal models remain necessary for causal experiments, but DSS, TNBS, DNBS/ethanol, and oxazolone models are chemically induced experimental colitis, not human UC. They capture selected epithelial-injury or immune features and differ in toxicity, inflammatory context, microbiota dependence, and recovery kinetics. Additional TCM syndrome-model procedures introduce further physiological perturbations that may independently alter food intake, stress, metabolism, and microbial ecology. Antibiotic depletion can also affect host immunity and epithelial metabolism independently of microbial removal, while FMT transfers phages, fungi, metabolites, and host-derived material together with bacteria. Interpretation should therefore separate disease-model biology from the imposed syndrome phenotype and replicate key findings in more than one experimental context when feasible.

For animal evidence, minimum bias assessment should document random sequence generation, allocation concealment where feasible, blinded outcome assessment, prespecified exclusions, sample-size justification, attrition, sex and age, housing/cage and co-housing strategy, litter or vendor effects, DSS/TNBS batch and exposure, sequencing batch, and whether microbiome analyses were performed on biologically independent samples. Microbiome studies should additionally report extraction procedures, negative controls, absolute as well as relative abundance when possible, and prespecified metabolite or functional endpoints. These items should accompany effect sizes rather than relying only on P values.

Negative findings also matter. Indigo naturalis, for example, aggravated oxazolone-induced experimental colitis, and gut-microbiota depletion attenuated this adverse effect, showing that a microbiota-dependent herbal action can be harmful rather than protective (Adachi et al., 2017). Such results argue against selecting only studies in which an intervention moves the microbiome toward a prespecified “healthy” direction. Null, bidirectional, and adverse microbiome outcomes are needed to define model dependence, therapeutic windows, and context-specific risk.

### 4.3 Human evidence, comparative context, and clinical safety

Clinical translation remains substantially less mature than the preclinical literature. A meta-analysis suggests that probiotics combined with TCM may improve selected clinical outcomes, but the contributing studies were heterogeneous in herbal composition, probiotic strain, dose, treatment duration, blinding, and outcome definitions (Hu et al., 2022). FMT has randomized-trial and guideline support as a microbiota-based strategy in UC, although current AGA guidance suggests against conventional FMT outside the context of clinical trials; donor selection, manufacturing, delivery route, antimicrobial-resistance screening, durability, and long-term safety remain important constraints (Zha et al., 2024; Peery et al., 2024; Belotserkovsky et al., 2024). Prebiotics and postbiotics also have emerging evidence (Sinopoulou et al., 2024; Wang S. et al., 2025). However, no direct randomized head-to-head trial identified in this review compared a standardized TCM preparation with FMT or with sodium-butyrate/SCFA therapy. Accordingly, similarities in microbial or metabolite endpoints should not be interpreted as comparative efficacy. Factorial or active-comparator trials are needed.

Human placebo-controlled evidence is available for several botanical interventions relevant to this pharmacological framework. Indigo naturalis produced dose-related short-term clinical and mucosal responses in an 8-week multicenter randomized trial, and a separate 2-week placebo-controlled study also showed clinical improvement (Naganuma et al., 2018; Uchiyama et al., 2020). Curcumin–QingDai improved clinical response, remission, and endoscopic outcomes at 8 weeks in a randomized placebo-controlled trial (Ben-Ho et al., 2024). *Andrographis paniculata* extract showed a significant clinical-response signal at 1,800 mg/day but not 1,200 mg/day (Sandborn et al., 2013). Curcumin 3 g/day added to mesalamine improved 4-week clinical and endoscopic outcomes (Lang et al., 2015), whereas 450 mg/day did not outperform placebo over 8 weeks (Kedia et al., 2017), illustrating that botanical effects cannot be generalized across dose or formulation. A 2025 multicenter randomized program in 143 patients also reported clinical, microbiome, and SCFA changes with three Chinese herbal strategies across disease-severity strata, including modified Wumei Pill plus mesalazine (Li N. et al., 2025). These studies are summarized in Table 3; none, by efficacy alone, establishes that the microbiota mediated the clinical response.

**TABLE 3:** Human interventional evidence for TCM-related and botanical therapies in ulcerative colitis.

Preparation	Design/comparator	N	Dose and concomitant therapy	Clinical outcome	Endoscopic/objective outcome	Follow-up	Safety
Indigo naturalis	Multicenter double-blind RCT; placebo	86	0.5, 1.0, or 2.0 g/day; background therapy permitted per protocol	Week-8 response: placebo 13.6%; indigo naturalis 69.6%, 75.0%, 81.0% across doses	Mucosal healing improved versus placebo; remission signal also observed	8 weeks	Mild liver dysfunction reported; trial stopped early after an external pulmonary arterial hypertension safety signal (Naganuma et al., 2018)
Indigo naturalis	Multicenter double-blind RCT; placebo	46 randomized; 42 analyzed	500 mg twice daily	Lichtiger index improved; response 82.6% vs. 26.3% reported in trial analyses	No prespecified endoscopic efficacy endpoint in the short trial	2 weeks	Mild headache more frequent; no serious PAH/intussusception during short exposure (Uchiyama et al., 2020)
Curcumin–QingDai	Randomized double-blind placebo-controlled phase II component	42	Enteric-coated Curcumin–QingDai (CurQD) 3 g/day; stable background therapy	Clinical response 85.7% vs. 30.7%; remission 50% vs. 8%	Endoscopic improvement 75% vs. 20%; composite objective/clinical endpoint 43% vs. 8%	8-week induction; responders followed to week 16	Adverse events comparable between groups in induction (Ben-Ho et al., 2024)
*Andrographis paniculata* extract (HMPL-004)	Randomized double-blind placebo-controlled trial	224	1,200 or 1,800 mg/day	Response 45% and 60% vs. 40% placebo; significant only for 1,800 mg/day	Mucosal healing 37.8% and 50.0% vs. 33.3% placebo; significant only for 1,800 mg/day	8 weeks	Overall adverse-event rates similar across arms (Sandborn et al., 2013)
Curcumin + mesalamine	Multicenter randomized double-blind placebo-controlled trial	50	Curcumin 3 g/day + continued optimized mesalamine	Remission 53.8% vs. 0%; response 65.3% vs. 12.5%	Endoscopic remission 38% vs. 0% among evaluated patients	4 weeks	Adverse events rare and comparable (Lang et al., 2015)
Low-dose curcumin + mesalamine	Randomized double-blind placebo-controlled trial	62	Curcumin 450 mg/day + mesalamine 2.4 g/day	No significant difference in remission or response	No significant difference in mucosal healing	8 weeks	No efficacy advantage; illustrates dose/formulation dependence (Kedia et al., 2017)
Hudi Enteric-coated capsules (HDEC)/Qingchang Wenzhong decoction (QCWZ)/modified Wumei Pill strategies	Three multicenter randomized controlled trials; mesalazine comparators	143 across 10 hospitals	Severity-stratified herbal therapy alone or with mesalazine	Mayo and systemic inflammatory outcomes were broadly similar across the herbal and mesalazine comparator strategies	Microbiome/SCFA changes were largest with modified Wumei Pill + mesalazine in severe UC; associations do not prove mediation	Trial-defined treatment follow-up; long-term safety was not established	No long-term comparative safety conclusion; standardized surveillance remains needed (Li N. et al., 2025)

Safety requires equal emphasis. Botanical identity, substitution/adulteration, contaminants, pesticide or heavy-metal exposure, extraction method, and batch-to-batch chemical variation can change both pharmacology and toxicity; commercial-product surveys demonstrate that authentication failures and compositional variability are not trivial (Ichim and Booker, 2021). Multicomponent formulas also complicate attribution of adverse events and create pharmacokinetic herb–herb and herb–drug interaction risks through transporters, metabolic enzymes, and microbiota-mediated biotransformation (Li M. et al., 2024). These concerns are particularly relevant when herbs are combined with corticosteroids, thiopurines, biologics, Janus kinase inhibitors, sphingosine-1-phosphate modulators, or other small molecules. For indigo naturalis, a nationwide Japanese survey recorded liver dysfunction, gastrointestinal events, headache, pulmonary arterial hypertension, and intussusception among exposed patients, underscoring the need for dose documentation and longitudinal surveillance rather than assuming safety from traditional use (Naganuma et al., 2019). Long-term ecological effects are also poorly characterized: repeated exposure to botanicals with antimicrobial activity could impose sustained selection on strains, microbial functions, or resistance genes even when short-term inflammation improves. Serial strain-resolved metagenomics, resistome assessment where relevant, and post-treatment sampling are therefore needed. Trials should prospectively define adverse-event attribution, liver and kidney monitoring, interaction surveillance, stopping rules, and post-treatment follow-up.

### 4.4 Numbered translational roadmap for individualized TCM–microbiome studies

A practical translational roadmap is:

1. Standardize the intervention: authenticate botanical species, document processing and extraction, establish chemical fingerprints and batch-release specifications, and quantify marker constituents and relevant contaminants.
2. Resolve exposure and causality: combine luminal and mucosal sampling, absolute microbial quantification, strain-resolved metagenomics, targeted metabolomics, and pharmacokinetics of parent compounds and microbial metabolites; use depletion/transfer/reconstitution and host-target perturbation when causal claims are made.
3. Prespecify response modifiers: genotype and host biology, baseline dysbiosis, disease activity and location, diet, antibiotics/probiotics, smoking, and concurrent drugs can all alter microbial ecology or herbal exposure. Baseline microbial features are already associated with subsequent therapeutic response in UC cohorts (Lloyd-Price et al., 2019; Halfvarson et al., 2017; Schirmer et al., 2018; Schirmer et al., 2024; Kulecka et al., 2025), while diet and medications are major determinants of microbiome structure (Karim et al., 2023). These factors should be measured rather than treated as noise.
4. Use clinically meaningful outcomes: prioritize endoscopic and histological remission, steroid-free remission, validated symptoms, and treatment durability; collect biospecimens at standardized time points to test whether microbial or metabolic changes precede rather than merely follow improvement.
5. Build safety into efficacy studies: prospectively monitor hepatic and renal function, herb–drug interactions, contaminants, antimicrobial-resistance genes where relevant, serious infections for microbiota-transfer procedures, and delayed adverse events or relapse.
6. Validate stratification: TCM syndrome categories and microbiome signatures should be locked before analysis and externally replicated. Interaction tests should determine whether they truly identify differential treatment benefit rather than *post hoc* subgroup correlations.

### 4.5 Plain-language translational summary for clinicians

For clinicians, the central message is simple. A higher relative abundance of *Lactobacillus* or *Akkermansia* after an herbal treatment does not prove that the microbiome has improved or that those organisms caused the clinical effect. Most TCM–microbiome evidence still comes from experimental colitis. Stronger causal evidence requires a consistent microbial function or metabolite change, loss or transfer of efficacy after microbiota manipulation, recovery with a defined strain or metabolite, and validation of the relevant host target. Human trials provide encouraging but preparation- and dose-dependent short-term signals, while long-term safety, chemical standardization, and interaction with established UC therapies remain limiting. At present, microbiota-directed TCM strategies should be studied as standardized, monitored adjuncts rather than assumed substitutes for evidence-based UC therapy.

## 5 Conclusion

The gut microbiota is an active component of UC pathogenesis and a plausible pharmacological interface, but it is not a single therapeutic target. TCM formulas, herbal fractions, purified compounds, vesicle-like particles, and delivery systems can affect microbial functions, epithelial defenses, and immune pathways through both direct host actions and microbiota-dependent mechanisms. The evidence base is nevertheless dominated by chemically induced experimental colitis and associative microbiome profiling. The most convincing studies combine microbiota-dependence tests with defined strain or metabolite manipulation and host-target perturbation; organoid responses or genus-level abundance shifts alone are insufficient. Human trials show that selected botanical preparations can produce short-term clinical and endoscopic signals, but efficacy varies by dose and formulation and long-term safety is incompletely characterized. Progress now depends on chemically standardized interventions, causal experimental designs, individualized assessment of baseline microbiome/diet/drug context, objective clinical endpoints, and prospective safety surveillance. TCM syndrome differentiation may contribute to stratification only if its reproducibility and predictive value are established prospectively across independent cohorts.
