# Supplementary material for: Microbiota–host crosstalk in ulcerative colitis: mechanistic evidence and translational prospects for traditional Chinese medicine
Source: Front Pharmacol. 2026 Oct 9;17:1956353. doi: 10.3389/fphar.2026.1956353 (PMC13691306; doi:10.3389/fphar.2026.1956353)
Supplement: Supplementary file 1 [file Supplementaryfile1.docx]

Supplementary Table S1. Representative pharmacological details for key multicomponent-formula studies cited in Table 1.

| **Representative preparation / study** | **Classical vs. modified; principal composition** | **Reported dose / route / duration** | **Chemical standardization / reproducibility information** | **Ref.** |
| --- | --- | --- | --- | --- |
| Sijunzi Decoction | *Panax ginseng; Atractylodes macrocephala; Poria cocos; Glycyrrhiza uralensis* | 200, 400, or 800 mg/kg/day by gavage for 7 days during 3% DSS exposure | UHPLC-QTOF-MS fingerprinting/chemical profiling reported | (Li H. et al., 2024) |
| Huangqin Decoction | *Scutellaria baicalensis; Paeonia lactiflora; Glycyrrhiza uralensis; Ziziphus jujuba* | 4.55 or 9.1 g/kg/day by oral gavage during 3.5% DSS exposure for 7 days; high dose used for mechanistic experiments | Mass spectrometry-based chemical profiling; antibiotic depletion and AhR antagonist controls included | (Li R. et al., 2026) |
| Baitouweng Decoction | *Pulsatilla chinensis; Coptis chinensis; Phellodendron chinense; Fraxinus rhynchophylla* | 3.38 or 6.75 g/kg/day orally on days 8–14 after 3.5% DSS induction | UHPLC-QTOF-MS quality-control analysis of extract | (Hu J. et al., 2025) |
| Shaoyao Decoction | *Paeonia lactiflora*-centered multicomponent formula; classical ingredients and preparation described in source study | 8, 16, or 32 g/kg/day for 7 days after model establishment | Chemical/metabolomic profiling reported; dose-response design used | (Zhen J. et al., 2024) |

Abbreviations: AhR, aryl hydrocarbon receptor; DSS, dextran sulfate sodium; UHPLC-QTOF-MS, ultra-high-performance liquid chromatography-quadrupole time-of-flight mass spectrometry.

**References**

Li, H., Pu, X., Lin, Y., Yu, X., Li, J., Bo, L., et al. (2024). Sijunzi decoction alleviates inflammation and intestinal epithelial barrier damage and modulates the gut microbiota in ulcerative colitis mice. *Front. Pharmacol.* 15, 1360972. doi: 10.3389/fphar.2024.1360972.

Li, R., Liao, X., Fu, X., Li, X., Liao, X., Cen, S., et al. (2026). Microbiota-driven tryptophan metabolism and AhR triggered intestinal stem cell differentiation: mechanisms of Huangqin decoction in ulcerative colitis repair. *Chin. Med*. 21, 33. doi: 10.1186/s13020-025-01302-y.

Hu, J., Chen, H., Zhu, L., Tong, Y., Cheng, C., Yan, G., et al. (2025). Baitouweng decoction modulates gut microbial production of indole-3-propionic acid and epithelial necroptosis to alleviate DSS-induced colitis in mice. *Chin. Med*. 20, 119. doi: 10.1186/s13020-025-01143-9.

Zhen, J., Li, Y., Zhang, Y., Zhou, Y., Zhao, L., Huang, G., et al. (2024). Shaoyao Decoction reduced T lymphocyte activation by regulating of intestinal flora and 5-hydroxytryptamine metabolism in ulcerative colitis. *Chin. Med*. 19, 87. doi: 10.1186/s13020-024-00958-2.
